# Assessment of the Response to Abdominal and Pelvic Computed Tomography Report Recommendations: A Single-Center, Retrospective, Chart Review Study

**DOI:** 10.7759/cureus.21190

**Published:** 2022-01-13

**Authors:** Shaza Alsharif, Ghalib Alasaad, Mohammed K Bukhari, Abdulaziz Sharkar, Mohammed Altaf, Shaymaa Milibari, Roaa Alsolaimani, Khalid M Alshamrani

**Affiliations:** 1 Research, King Abdullah International Medical Research Center, Jeddah, SAU; 2 College of Medicine, King Saud Bin Abdulaziz University for Health Sciences, Jeddah, SAU; 3 Medical Imaging, Ministry of National Guard - Health Affairs, Jeddah, SAU; 4 Neurology, College of Medicine, King Saud Bin Abdulaziz University for Health Sciences, Jeddah, SAU; 5 Department of Quality, King Faisal Residential City Clinic, Jeddah, SAU; 6 College of Medicine, King Abdulaziz University, Jeddah, SAU; 7 College of Applied Medical Sciences, King Saud Bin Abdulaziz University for Health Sciences, Jeddah, SAU

**Keywords:** abdominal and pelvic computed tomography, medical imaging informatics, health informatics, compliance, follow-up recommendations, ct scan reports, radiologist recommendation, radiology report

## Abstract

Objectives

The radiology report is the primary form of communication between the radiologists and referring clinicians. It is a structured document containing several key components pertaining to the interpretation of radiological examinations and may require the addition of follow-up imaging recommendations to optimize patient outcomes. This study aims to determine whether follow-up imaging recommendations are being acknowledged and acted upon by referrers.

Methods

This retrospective study was conducted at a single tertiary hospital. Prerecorded BESTCare data of patients who underwent abdominal and pelvic computed tomography (CT) scans between October 1, 2017, and December 31, 2017, and received recommendations for further evaluation were collected after obtaining ethical approval from the local authority. Data of patients younger than 14 years old, patients who did not receive a recommendation, and patients who had CT scans that were uploaded to the BESTCare system but were performed outside the institution were excluded. The collected data were recorded in a password-protected Microsoft Excel file for further analysis.

Results

A total of 523 report recommendations from 422 abdominal and pelvic CT reports were analyzed. The most common organs indicated for CT scan evaluation were the breast (N = 54, 10.33%), kidney (N = 46, 8.80%), lymph node (N = 36, 6.88%), and colon (N = 33, 6.31%). The most common type of further evaluation recommended was further imaging (N = 410, 78.39%). A total of 278 (53.15%) recommendations were not performed, with 199 (71.58%) not having a documented rationale for noncompliance.

Conclusion

The majority of the follow-up imaging recommendations to ordering physicians were not carried out. This study highlights the need for notification and audit systems to monitor compliance with follow-up recommendations. Improving the communication between radiologists and referring physicians is key to optimizing patient healthcare.

## Introduction

The radiology report is a structured document containing several key components pertaining to the interpretation of radiological examinations that can be used to narrow the differential diagnosis and may require the addition of follow-up imaging recommendations to optimize patient outcomes and care [1,2]. It is the primary form of communication between radiologists and referring physicians toward integrating clinical information with the radiological findings and data from other departments [3-5]. The need for effective communication has greatly increased owing to the existence of a wide spectrum of medical subspecializations, the advancement of medical technologies, and the significance of findings from many departments to reach a diagnosis and commence management [6,7]. Furthermore, the increased complexity of recent medical imaging techniques stresses the need for accurate presentation and description of findings [5]. Radiology reports may require the addition of recommendations that could assist in clearing up any uncertainties, confirming a diagnosis, or reporting incidental findings, and therefore, such recommendations may greatly impact the healthcare services delivered to the patient [3]. A study conducted in Waikato Hospital in Hamilton, New Zealand, reported that the likelihood of further investigation of adrenal incidentalomas according to guidelines increased as a result of radiology report recommendations [8]. It is expected that these recommendations will be acknowledged by the referring physicians, as any disregard for radiology report recommendations may negatively impact the quality of the service. Low adherence to recommendations regarding follow-up imaging may lead to subsequent poor healthcare delivery, avoidable unnecessary tests, and liability for legal action [9,10].

Prior studies have discussed the response to radiology report recommendations in various fields and the rate at which the recommendations were carried out. However, to the best of our knowledge, there are no former studies specifically directed toward the response to abdominal and pelvic computed tomography (CT) report recommendations. The aim of this retrospective study is to assess referrers' responses to follow-up radiology report recommendations. We further highlight the potential reasons for the variation in the response to recommendations with the goal of improving communication between referring physicians and radiologists, which will lead to an improvement in the quality of service delivered to patients.

## Materials and methods

Study design and sampling technique

This study implemented a retrospective, chart review study design and a non-probability convenient sampling technique to collect the radiology reports of all abdominal and pelvic CT performed between October 1, 2017, and December 31, 2017, in King Abdulaziz Medical City, Ministry of National Guard - Health Affairs (MNG-HA), Jeddah City, Kingdom of Saudi Arabia (KSA).

Inclusion and exclusion criteria

The subjects included in this research were all adults who underwent abdominal and pelvic CT and received a recommendation for further evaluation. Patients under 14 years of age, patients who did not receive a recommendation, and patients who had CT scans that were uploaded to the BESTCare system but were performed outside the institution were excluded.

Data collection

Access to patient documents and electronic files was through the electronic medical record system (BESTCare). The collection of the data was self-administered by four medical students into a password-protected Microsoft Excel sheet after obtaining institutional review board (IRB) approval from the local authority. Patients were categorized according to age, gender, the type of recommendation received, the referral department and indication for the CT scan, whether the recommendation was in the same organ system imaged, and whether the recommendation was done or not.

Ethical consideration

The ethics committee of King Abdullah International Medical Research Center issued institutional review board (IRB) approval #SP18/331/J. The data were anonymized, and confidentiality of patient information was maintained throughout the project.

Statistical analysis

The data were analyzed using the statistical package IBM SPSS version 24 and Minitab version 17. Initial descriptive analysis (i.e., mean and standard deviation and percentages) of demographical characteristics was generated. The Chi-square test of independence was used to determine the relationship between a) the recommendations that were done and whether they were done in the same system as the CT indication and b) the recommendations that were done and the type of further evaluation recommended. A p-value of less than 0.05 was considered significant.

## Results

A total number of 423 abdominal and pelvic CT reports were included in this study from 413 patients. The reports contained a total of 550 recommendations, of which 523 recommendations were analyzed. The remaining 27 recommendations were excluded due to missing information. The patients' ages range from 14 to 103 years (mean ± SD = 55.14 ± 17.39), 244 (59%) of which are females and 169 (41%) are males. Patients undergoing abdominal and pelvic CT were referred from different departments. The majority of reports with radiology recommendations were referred from the Adult Medical Oncology Department (124/413, 30.02%) and the Emergency Department (76/413, 18.40%), followed by the Gastroenterology Department (31/413, 7.51%) and the General Surgery Department (30/413, 7.26%) (Table 1).

**Table 1:** Patient Demographics

Demographic Variables	Frequency (N = 413)	Percentage
Age (mean ± SD)	55.14 ± 17.39	
Gender		
Male	169	41%
Female	244	59%
Referral Department		
Adult Medical Oncology	124	30.02%
Emergency Medicine	76	18.40%
Gastroenterology	31	7.51%
General Surgery	30	7.26%
Internal Medicine	23	5.57%
Urology	23	5.57%
Radiation Oncology	15	3.63%
Neurology	14	3.39%
Thoracic Surgery	10	2.42%
Others	67	16.22%

**Figure 1:**
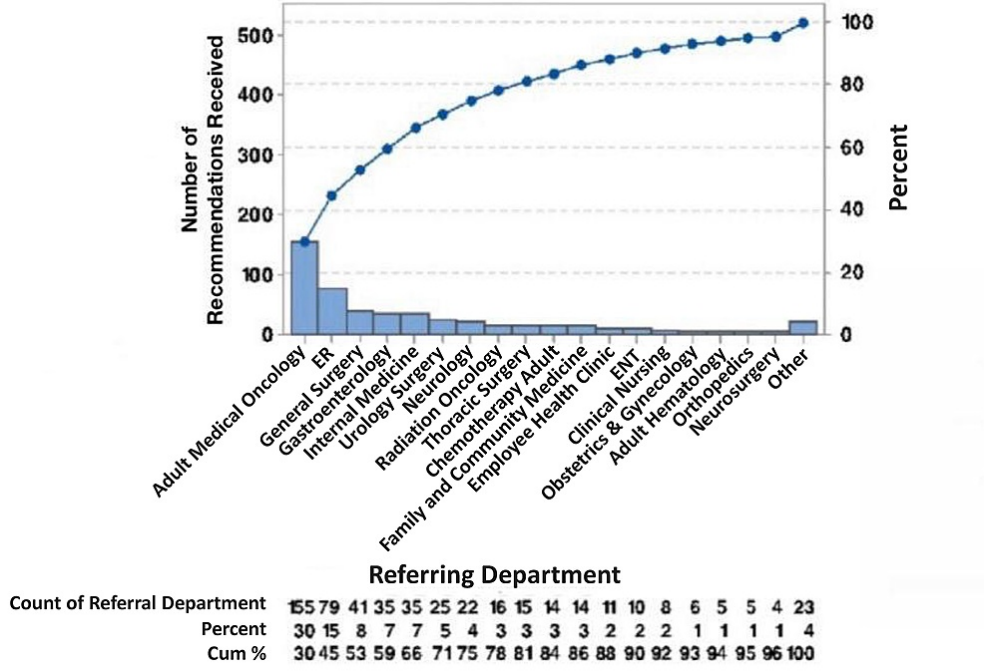
Recommendations Received by the Referring Departments

The only exceptions are that the General Surgery Department received more recommendations than the Gastroenterology Department and the Neurology Department received more recommendations than the Radiation Oncology Department (Figure 1, Table 2).

**Table 2:** Recommendations Received by the Referring Departments

Row Labels	Number of Recommendations Received by the Referring Departments	Percentage
Adult Medical Oncology	155	29.64%
Emergency Medicine	79	15.11%
General Surgery	41	7.84%
Gastroenterology	35	6.69%
Internal Medicine	35	6.69%
Urology Surgery	25	4.78%
Neurology	22	4.21%
Radiation Oncology	16	3.06%
Thoracic Surgery	15	2.87%

The patients whose recommendations were analyzed (some of whom received more than one recommendation) underwent a CT scan due to various medical indications. The most common organs indicated for CT scans were the breast (54/423, 12.77%), kidney (46/423, 10.87%), lymph node (36/423, 8.51%), and colon (33/423, 7.80%) (Table 3).

**Table 3:** Indication for Abdominal and Pelvic CT

Row Labels	Number of CT Indication	Percentage
Breast	54	12.77%
Kidney	46	10.87%
Lymph node	36	8.51%
Colon	33	7.80%
Liver	25	5.91%
Bowel	24	5.67%
Lungs	21	4.96%
Abdomen	18	4.26%
Malignancy	17	4.02%
Abdominal wall	17	4.02%

**Figure 2:**
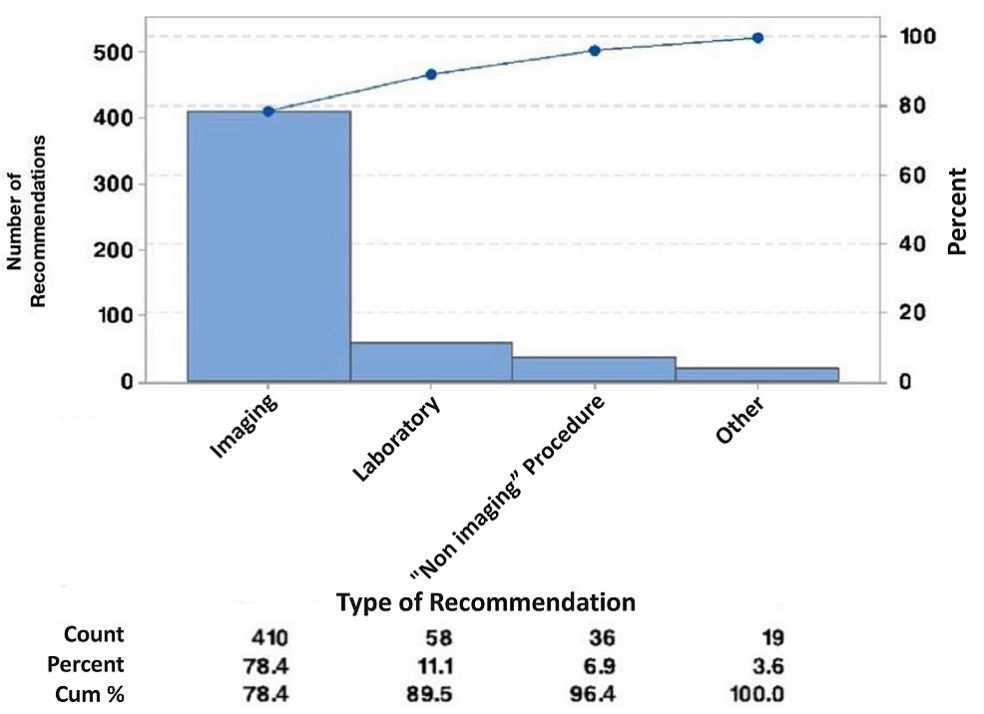
Type of Further Evaluation Recommended

Sorted into different categories, the most common types of further evaluation recommended were further imaging (410/523, 78.39%), further laboratory evaluation (60/523, 11.47%), non-imaging procedures (37/523, 7.07%), and specialty referrals (16/523, 3.06%) (Figure 2, Table 4).

**Table 4:** Type of Further Evaluation Recommended

Row Labels	Type of Recommendation Received	Percentage
Imaging	410	78.39%
Laboratory	60	11.47%
Non-imaging procedure	37	7.07%
Specialty referral	16	3.06%
Grand total	523	100%

The renal organ system received the most recommendations for further evaluation (111/523, 21.23%), followed by the hepatobiliary system (101/523, 19.31%) and the genitourinary system (88/523, 16.83%) (Table 5).

**Table 5:** Organ System Recommended to be Evaluated Further

Organ System Examined	Number of Organ System Examined	Percentage
Renal	111	21.23%
Hepatobiliary	101	19.31%
Genital	88	16.83%
Other	63	12.04%
Gastrointestinal	50	9.56%
Respiratory	32	6.12%
Musculoskeletal	42	8.03%
Lymphatic system	22	4.21%
Pulmonary	13	2.49%
Cardiovascular	1	0.19%
Grand total	523	100%

There were 158/523 (30.21%) recommendations in the same organ system as the indication for the abdominal and pelvic CT, whereas 358/523 (68.45%) of the recommendations were not in the same organ system, and 7/523 (1.34%) of the recommendations were not applicable to the criteria (Table 6).

**Table 6:** Relationship of Organ System Recommended to be Evaluated Further to the CT Indication

Is the Recommendation in the Same Organ System as the CT Indication?	Number	Percentage
No	358	68.45%
Yes	158	30.21%
Inconclusive	7	1.34%
Grand total	523	100%

Of the 523 recommendations studied, 278 (53.15%) were not performed and 245 (46.85%) of the recommendations were performed. The recommendations performed within six months were 202 (38.62%), and 321 (61.38%) were not performed within six months (Table 7).

**Table 7:** Number of Recommendations Performed and Not Performed Within Six Months

Done Within Six Months?	Number	Percentage
No	321	61.38%
Yes	202	38.62%
Grand total	523	100%

The number of non-performed recommendations was 278 (53.15%), whereas the number of performed recommendations was 245 (46.85%), of which 43 (17.55%) recommendations were not performed within a six-month period (Table 8).

**Table 8:** Number of Recommendations Performed

Recommendation Done?	Number	Percentage
No	278	53.15%
Yes	245	46.85%
Grand total	523	100%

**Figure 3:**
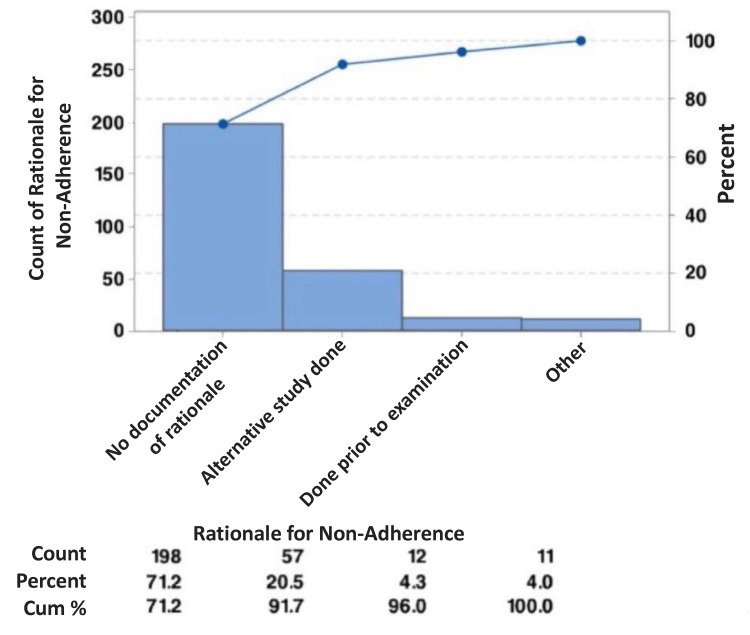
Rationale for Not Performing the Recommendation

It should be noted that some recommendations were for follow-up of certain findings after a six-month period. Of the 278 non-performed recommendations, 199 (71.58%) did not have a documented rationale, and 58 (20.86%) were not performed because an alternative study was done. Additionally, 12 (4.32%) were not performed because a study was already performed before the CT scan (Figure 3, Table 9).

**Table 9:** Rationale for Not Performing the Recommendation

Reason for Not Performing the Recommendation	Number	Percentage
No documentation of rationale	199	71.58%
Alternative study done	58	20.86%
Done prior to examination	12	4.32%
Patient no show	8	2.88%
Appointment postponed	1	0.36%
Grand total	278	100%	

The recommendations that were done were compared with whether the recommendations were in the same system as the CT indication, with a likelihood ratio of 4.106 (DF = 1, P = 0.043), indicating a significant association between the recommendations performed and whether they were performed in the same system as the CT indication. The recommendations that were done were also compared with the type of further evaluation recommended, with a likelihood ratio of 17.841 (DF = 3, P = 0.001), indicating a significant association between the recommendations performed and the type of further evaluation recommended.

## Discussion

The primary aim of the study was to assess the effectiveness of radiology report recommendations by analyzing the execution and/or response of the referring departments to the recommendations provided in the radiology report. The study findings revealed that 53.15% of the recommendations were not carried out. The secondary objective was to identify the rationale behind not following through on the recommendations. Most of the recommendations did not contain a specific rationale for why they were disregarded (N = 199, 71.58%).

A study at an academic children's hospital sought to determine the proportion of radiology report recommendations that were acknowledged by referring physicians [3]. The study analyzed 453 reports from 370 patients and found that 140 recommendations were not executed and only 40% were mentioned in the clinical notes. The study had a lower percentage of disregarding of recommendations compared to our study. Similarly, Intermountain Medical Center researchers found that of the 1000 CT pulmonary angiographic studies received, 9.9% showed incidental pulmonary nodules requiring additional follow-up [11]. Only 29% of these studies were followed up, indicating a lower rate of recommendation adherence than our study, which considered all recommendations equally. A study conducted at Staten Island University Hospital aimed to determine the number of incidental findings and the need for a follow-up of abdominal CT reports at a pediatric trauma center. The study analyzed 418 incidental findings from 345 patients and found that 60 might require outpatient monitoring. The study also concluded that approximately one-third of the patients had radiological findings not attributed to their injuries, and one-third of the patients needed further workup [12].

Another study examined whether expressions of doubt and further imaging recommendations affected the follow-up rate. The study reviewed 250 outpatient reports and found that 92 (36.8%) lacked a timely response, whereas, in our study, 38.6% of the total recommendations were performed within six months. The study also found that further imaging recommendations had a higher chance of a lack of follow-up [13]. Another study analyzed the frequency of additional imaging recommendation acceptance. The study analyzed 430 scans from a tertiary medical center and identified further imaging recommendations in 67 cases (15.58%). In contrast, in our study, further imaging recommendations were the majority (78.39%). The recommendations were not completed in 43 cases (64.18%), indicating a percentage higher than the one reported in our study. No rationale was documented in 38.6% of the recommendations not performed, whereas, in our study, 71.58% of recommendations not performed did not have a documented rationale. The study also found that, contrary to concerns of auto referral to further imaging by radiologists, the rate of these referrals was less than that of prior studies [14].

The study may have been limited by the relatively short time frame of abdominal and pelvic CT reports, from October 1, 2017, to December 31, 2017. Moreover, the study was conducted at a single tertiary care hospital. Another possible limitation may have been that the only way to document the rationale for not performing recommendations was to add notes to the patient's medical record. Some of the recommendations were scheduled for six months or longer after the CT, which affected our analysis of how timely the recommendations would have been implemented.

## Conclusions

In conclusion, by a relatively slim margin, the majority of recommendations were not performed, and in most cases, there was no documented rationale for the non-performance of the recommendations. There are some limitations to following the recommendations stated in the radiology report. However, there are certain measures that can be taken to prevent the poor utility of the recommendations. The attitude of physicians toward following these recommendations as a medical obligation enhances the chances that a proper response will ensue. A possible solution method to improve the quality of radiology reports is by implementing guidelines or advocating the use of a more structured style of reporting. Verbal and electronic notification can be associated with increased follow-up of recommendations. Audit systems or key performance indicators (KPIs) can also be implemented to improve recommendation compliance.
